# A Military Audio Dataset for Situational Awareness and Surveillance

**DOI:** 10.1038/s41597-024-03511-w

**Published:** 2024-06-22

**Authors:** June-Woo Kim, Chihyeon Yoon, Ho-Young Jung

**Affiliations:** Department of Artificial Intelligence, Kyungpook National University, Daegu, 41566 South Korea

**Keywords:** Developing world, Databases

## Abstract

Audio classification related to military activities is a challenging task due to the high levels of background noise and the lack of suitable and publicly available datasets. To bridge this gap, this paper constructs and introduces a new military audio dataset, named MAD, which is suitable for training and evaluating audio classification systems. The proposed MAD dataset is extracted from various military videos and contains 8,075 sound samples from 7 classes corresponding to approximately 12 hours, exhibiting distinctive characteristics not presented in academic datasets typically used for machine learning research. We present a comprehensive description of the dataset, including its acoustic statistics and examples. We further conduct a comprehensive sound classification study of various deep learning algorithms on the MAD dataset. We are also releasing the source code to make it easy to build these systems. The presented dataset will be a valuable resource for evaluating the performance of existing algorithms and for advancing research in the field of acoustic-based hazardous situation surveillance systems.

## Background & Summary

Recent breakthroughs in deep learning have resulted in substantial advancements across diverse domains including audio classification. Generally, the creation of trustworthy artificial intelligence (AI) models hinges on the utilization of datasets characterized by high quality and representativeness^1–3^. Poor-quality datasets, facing many issues such as errors, inconsistencies, or missing values, have tend to yield AI models that are both inaccurate and biased. Furthermore, AI models trained on datasets that fail to represent the real world may exhibit poor performance when they are confronted with real-world applications. Moreover, limited data and a lack of diversity can also lead the AI models to be oversimplified and biased. To address the concerns raised above, it is essential to carefully construct high-quality datasets.

Audio classification is the task of automatically assigning audio signals into predefined categories. It is a rapidly expanding field with many real-world applications such as urban sound planning^4–7^, bioacoustic monitoring as well as healthcare^8–10^, multimedia event detection^11–14^, large-scale event discovery, surveillance^15–19^, and noise monitoring^20^ for industrial purposes. Furthermore, the audio classification research community has experienced significant growth in recent years, driven by the Detection and Classification of Acoustic Scenes and Events (DCASE) Challenge^21–24^. This challenge assisted the research and evaluation of publicly available common audio datasets, which has played a pivotal role in advancing the field of audio classification. Moreover, recent breakthroughs in deep learning have also facilitated the extension of audio classification to human-computer interaction tasks such as keyword spotting^25^, spoken language understanding^26^, and speech-based sentiment analysis^27^.

On the one hand, AI-based audio hazard detection systems are gaining traction in diverse applications and industries, leveraging AI to analyze audio data and detect potential hazards or dangers in the environment^18,19^. Despite the abundance of publicly available audio data, datasets suitable for training acoustic-based hazardous situation surveillance systems remain scarce. For instance, while siren-based^28^ systems can be helpful in traffic flow control and emergency response time reduction, they are ineffective in mass shooting or explosion scenarios. Although the AudioSet^29^ dataset has unprecedented data volume and diversity (approximately 2 million audio samples and 527 classes), it remains insufficient for training acoustic-based hazard detection systems due to its lack of dangerous audio signals. Besides, the classification of potentially dangerous acoustic events solely based on gunshot and breaking glass audio samples^30^ exhibits insufficient and requires further exploration of broader acoustic indicators. Therefore, developing a robust acoustic hazardous situational awareness and surveillance system requires a specific, varied, and wide range of dangerous audio signals such as explosions, shelling, and gunshots.

**Fig. 1:**
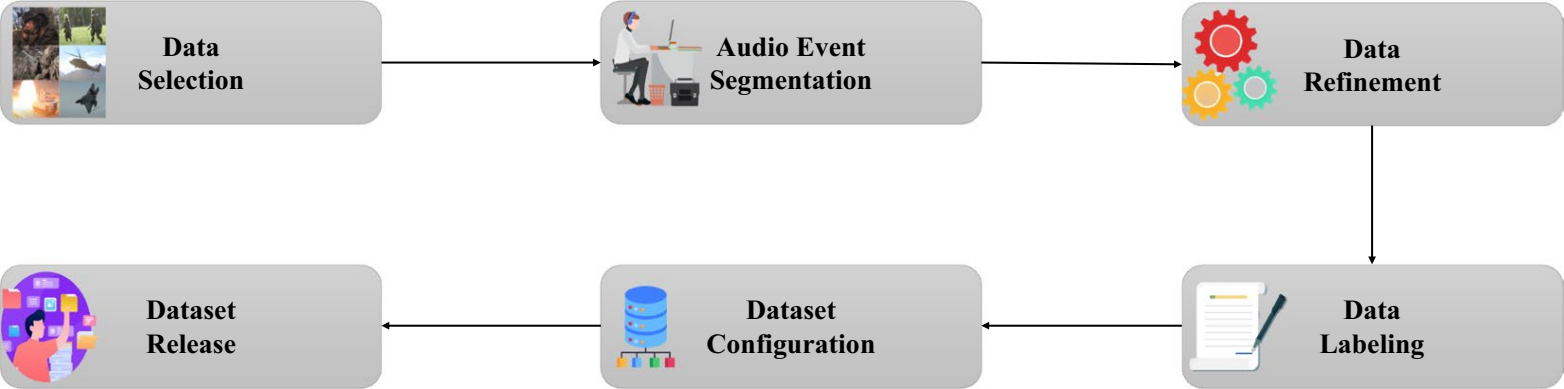
Illustration of MAD dataset collection procedure.

In this paper, we address these challenges by building and introducing the MAD for situational awareness and surveillance. Fig. 1 illustrates the overall data collection framework for the MAD dataset, which can be summarized as follows:

- Data selection: Identify the audio content types for the seven military-related categories.
- Audio event segmentation: Identify the start and end times of each audio event.
- Data refinement: Refine the data using a variety of techniques, such as formatting, resampling, and extracting.
- Data labeling: Label all audio events into one of the seven predefined classes.
- Data configuration: Randomly split all the data into train/test sets with an approximately 9:1 ratio.

**Fig. 2:**
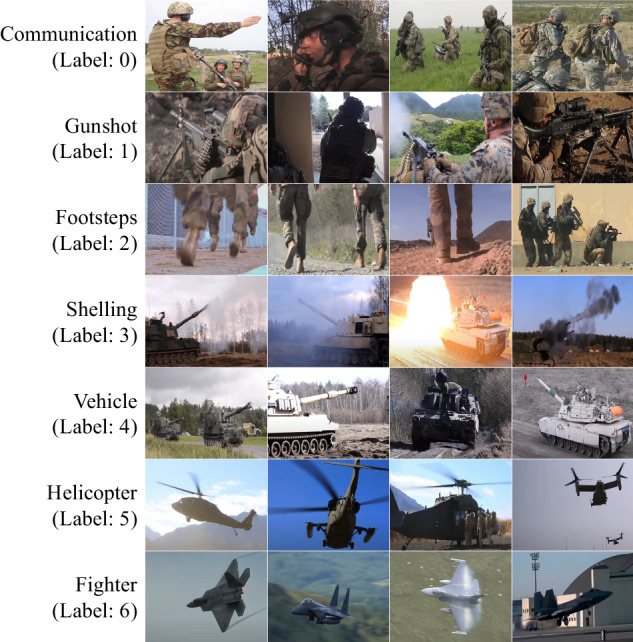
Demonstration of the overall MAD dataset.

As shown in Fig. 2, the MAD dataset is derived from diverse military videos, consequently categorized into seven classes, comprising 8,075 sound samples totaling approximately 12 hours of audio. In contrast to academic and research datasets commonly employed for machine learning research, the MAD dataset exhibits distinctive characteristics that facilitate the detection of acoustic-based hazard situations, such as gunshots, shelling or explosions, and fighter jets. We also present an overall description of the dataset including its acoustic statistics and examples.

In addition, we conduct a comprehensive evaluation of various deep learning algorithms for audio classification on the MAD dataset. Specifically, we benchmark the sound classification accuracy of several popular models widely used in the audio classification domain, including ResNet^31^, EfficientNet^32^, CNN-N (PANNs)^33^, and AST^10,34^. We also release the source code for the reproducibility of the paper and to help other researchers easily build such systems.

The proposed MAD dataset can be used to evaluate the performance of existing acoustic hazardous situation surveillance algorithms on a variety of hazards and environments, enabling the identification of strengths and weaknesses of various deep learning algorithms and guiding the development of improved methods. Overall, the MAD dataset represents a valuable resource for the research community working to develop and improve acoustic hazardous situation surveillance systems. We believe that the MAD dataset can be a valuable asset to the field of audio-based hazard detection, contributing to a safer world.

## Methods

This section discusses the methodology used to collect the MAD dataset as described in Fig. 1.

### Data selection

To gather realistic and dangerous audio data suitable for hazard detection systems, we first considered the appropriate data environment. In general, military activities, such as gunshots and explosion sounds, provide a rich source of audio data that can fulfill these requirements. Therefore, we decided to collect audio data related to military activities and further categorize it into seven distinct classes. Fig. 2 represents the overall classes of the MAD dataset: communication, gunshot, footsteps, shelling, vehicle, helicopter, and fighter. This comprehensive categorization ensures that the dataset encompasses a wide range of military-relevant sounds.

We subsequently queried YouTube for military activity videos. To collect more specific audio data related to military activities, we configured the search to identify audio event segments for predefined classes, leveraging videos collected from diverse countries and real-world environments. In particular, our dataset primarily comprises military-related training and education videos, which provide practical scenarios enriched with authentic background noises, such as wind, behavioral sounds, and shouting. Note that we intentionally excluded audio clips from hypothetical scenarios, such as games or simulations, to ensure the authenticity and practical acoustic-based hazard detection applicability of the dataset.

### Audio event segmentation

Once we select appropriate videos containing audio related to military activities, we then segmented each audio event to ensure its relevance and suitability for training deep learning models. Considering the vast range of audio event durations and the substantial time and computational resources required to train deep learning models on long audio sequences, we standardized the duration of all audio events to below 10 seconds. Despite the extensive time and effort involved in audio event segmentation, this strict process is crucial for constructing a high-quality dataset. To maintain the high-quality data, we thoroughly verified each segmented audio event with five annotators.

### Data refinement

We refine the collected data by formatting, resampling and extracting as described below.

#### Formatting

Following the data collection procedures outlined earlier, we downloaded the MP4 format videos and performed audio event segmentation. Nevertheless, since the primary objective of this study is to collect audio data, the videos are converted to WAV format. As part of the data preparation process, we utilized Python yt_dlp library for downloading YouTube videos and FFmpeg tool for subsequent audio extraction and formatting. This approach yielded the proposed MAD dataset of audio files with consistent characteristics of 192 Kbps bitrate, 48 kHz sampling rate, and a single audio channel. This allows for minimizing the space required for data storage without compromising audio quality.

#### Resampling

Sampling rate determines the frequency at which the audio signal is sampled and digitized, and varies depending on the recording device and software used, potentially introducing inconsistencies in the dataset. To ensure data consistency, we resampled all audio samples to a unified sampling rate of 16 kHz with the Python Librosa library. Resampling audio samples to a consistent sampling rate can be an effective technique for improving the performance of AI models in audio classification. This standardization process ensures that AI models are trained on more uniform data, leading to better generalization performance and reduced overfitting. Additionally, resampling facilitates the comparison of various models that utilize different sampling rates, providing a more comprehensive evaluation of their performance.

#### Extracting

We leveraged the Python Librosa and SoundFile library which are widely used tools for audio signal analysis for cropping audio data events following their corresponding segmentation labels. In other words, each audio sample was extracted by slicing the audio file from the start to the end time of its corresponding segmentation label. We subsequently saved these extracted and segmented audio events derived from the converted audio file obtained from the original video.

### Data labeling

Audio data labeling is the process of assigning predefined labels to audio clips to indicate the categories of specific sound events. In general, this process can be accomplished manually or using automated tools. While automated tools can annotate large amounts of data quickly and cheaply, they may not be as accurate as human annotators. To ensure accurate labeling, we chose a manual approach, employing human annotators to label every audio clip. Considering that each audio clip may contain a variety of sounds, our labeling process focused on identifying and annotating the most prominent or dominant sound class (i.e., clear or loud sound) within each clip. Subsequently, we subjected all labeled audio samples to a strict review and validation process, involving five annotators who independently voted on each label. This double-checking procedure improved the reliability of the audio data.

### Data configuration

Typically, the training set is designated for training machine learning models, while the test set serves as the means to evaluate the performance of the trained models on previously unseen data. In other words, splitting the data into training and test sets is essential to avoid overfitting, where the model becomes too specialized to the training data and cannot generalize well to new data. Moreover, evaluating the model on the test set which has not been encountered during training yields a more precise and reliable assessment of its overall performance.

To enable comparable experiments on the released dataset, we define train and test splits. We randomly split the whole dataset into training and test sets using the scikit-learn(could you make this “scikit-learn” word with the same format as “yt_dlp”, “FFmpeg” and “Librosa” format?) library in Python. The split was set with a ratio of 90% for training and 10% for test sets. Note that all audio events are divided into either training or test sets at the video level, thus audio events derived from the same video should belong to the same split. In other words, splitting the data into training and test sets is essential to avoid overfitting, where the model becomes too specialized to the training data and cannot generalize well to new data. Moreover, evaluating the model on the test set which has not been encountered during training yields a more precise and reliable assessment of its overall performance.

### Data release

In this section, we present a specific description of the MAD dataset, including its statistics and acoustic examples.

**Fig. 3:**
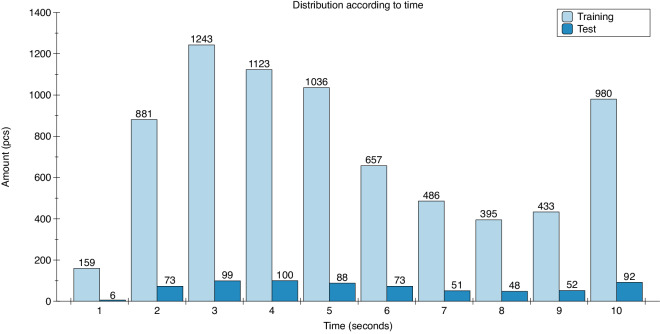
Bar chart illustrating the distribution of training and test sets according to various times.

As shown in Table 1, we have gathered 8,075 audio events from various videos, corresponding to 12.05 hours. Following the training/test set split, the MAD dataset was split into a training set of 7,393 audio samples (10.98 hours), and the test set contains 682 audio samples (1.07 hours). All the audio events were meticulously labeled and categorized, ensuring the dataset’s high quality and suitability for deep learning-based audio classification tasks. All audio events within the MAD dataset have durations between 1 and 10 seconds, with a distribution illustrated in Fig. 3. Notably, both the training and test sets exhibit the highest sample count at 3 seconds, while 1 second has the lowest.

**Table 1:** Overview of total data volume and time on MAD dataset.

	train	test	sum
sample size (pcs)	7,393	682	8,075
total time (hours)	10.98	1.07	12.05

Table 2 provides a detailed breakdown of the data statistics for each class within the MAD dataset, including the number of samples, percentage, and total time duration across training and test sets. The Gunshot class stands out with the highest percentage, accounting for 1,589 (21.23%, 2.35 hours). Conversely, the Footsteps class demonstrates the lowest percentage, with 921 (11.41%, 1.19 hours) instances.

**Table 2:** Overall details of the MAD dataset.

class	number of audio samples (total time, ratio)		
	train	test	sum
Communication	1,061 (1.79H, 14.35%)	89 (0.15H, 13.05%)	1,150 (1.94H, 14.24%)
Gunshot	1,589 (2.15H, 21.49%)	125 (0.20H, 18.33%)	1,714 (2.35H, 21.23%)
Footsteps	827 (1.07H, 11.19%)	94 (0.12H, 13.78%)	921 (1.19H, 11.41%)
Shelling	1,084 (1.04H, 14.66%)	89 (0.09H, 13.05%)	1,173 (1.13H, 14.53%)
Vehicle	987 (1.57H, 13.35%)	136 (0.23H, 19.94%)	1,123 (1.80H, 13.91%)
Helicopter	939 (1.79H, 12.70%)	70 (0.13H, 10.26%)	1,009 (1.92H, 12.50%)
Fighter	906 (1.57H, 12.26%)	79 (0.15H, 11.59%)	985 (1.72H, 12.20%)
Total	7,393 (10.98H, 91.55%)	682 (1.07H, 8.45%)	8,075 (12.05H, 100%)

**Fig. 4:**
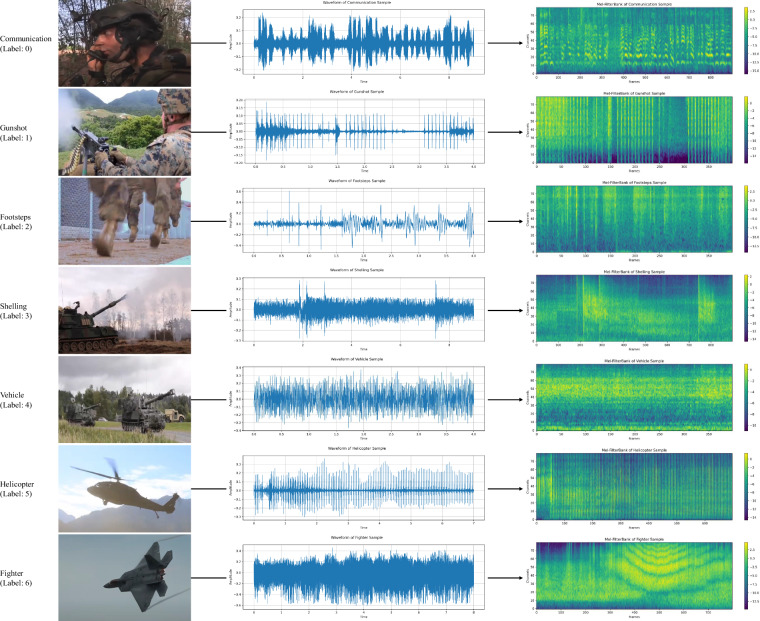
The overall pre-processing task for converting waveform to Mel filterbank.

Fig. 4 provides illustrative visualizations for each of the audio classes within the MAD dataset. This figure depicts the raw waveforms and Mel filterbanks for each class. From top to bottom, the classes are posted in the following order: communication, gunshot, footsteps, shelling, vehicle, helicopter, and fighter. The middle part of each row shows a randomly sampled raw waveform from the corresponding audio class. Furthermore, the right part of each row visualizes the Mel filterbank that will be used as the input speech features for the deep learning model. The Mel filterbank representations effectively capture the spectro-temporal characteristics of the audio signals, providing insights into the distinctive acoustic features of each class. Details of the pre-processing for deep learning model input will be provided in the Technical Validation section.

## Data Records

The MAD dataset is available for download at Figshare^35^. To facilitate dataset utilization and promote reproducibility, we provide comprehensive instructions within the README file at the provided URL, detailing the MAD dataset processing pipeline. This includes guidance on downloading YouTube videos using the supplied metadata annotation files. Readers can leverage the supplied metadata annotations to efficiently download relevant YouTube videos for research purposes. Besides, we divided all audio events into training and test folders based on the corresponding label files. The audio clips are in WAV format with a total data size of approximately 1.4 GB. The label files are in CSV format and include data paths, audio event labels, video titles, and corresponding URLs. We only provide the annotation files (CSV files); details on audio feature extraction for the deep learning model are discussed in the next section. The source to download the YouTube videos and process as well as extract the audio events for building models can be found in the GitHub repository (https://github.com/kaen2891/military_audio_dataset). Furthermore, the source code for training AI models is also available, which will be presented in the Code Availability section.

## Technical Validation

To evaluate the performance of the MAD dataset constructed in this paper, we conduct a comprehensive analysis of various deep learning-based algorithms for audio classification. Specifically, we compare the sound classification accuracy of several popular neural networks, including ResNet^31^, EfficientNet^32^, CNN-N (PANNs)^33^, and AST (Audio Spectrogram Transformer)^10,34^, which are widely used in the audio classification domain and have demonstrated strong performance on various benchmark datasets.

### Pre-processing

To train deep learning models with audio clips, we converted the audio waveforms to log Mel filterbank features using the TorchAudio library in Python. Specifically, we used a window size of 25 milliseconds and an overlap size of 10 milliseconds to extract 80-dimensional log Mel filterbank features from the audio waveforms. We ensured that all audio samples had the same length of 10 seconds, corresponding to 998 frames. For audio events shorter than 10 seconds, we performed zero padding with a fade in/out operation until the desired length. Moreover, we applied standard normalization to the spectrograms to ensure zero mean and unit variance. This process of converting audio waveforms to log Mel filterbank features is a common practice in audio classification tasks, as it allows the deep learning model to focus on the most relevant information in the audio signal. The normalization step further ensures that the features are evenly distributed, which can improve the performance of the model.

### Deep learning models

Table 3 provides a comprehensive overview of the various deep learning architectures used in this study, encompassing ResNet^31^, EfficientNet^32^, CNN-N (PANNs)^33^, and AST^10,34^. These architectures were chosen to represent a range of model complexities, with ResNet being represented by four distinct types (ResNet18, ResNet34, ResNet50, and ResNet101), EfficientNet being represented by three types (EfficientNet-B0, EfficientNet-B1, and EfficientNet-B2), and CNN-N being represented by three types (CNN6, CNN10, and CNN14). In addition, AST models include fine-tuning with AST^34^ and AST-Patch-Mix^10^ (AST model with Patch-Mix augmentation), with the former training with Cross Entropy loss between the input audio events and labels while the latter includes an augmentation method at the patch level.

**Table 3:** Overall details of the various architectures, including number of parameters and pretraining datasets.

architecture	parameters	pretrain
ResNet18	11.7M	
ResNet34	21.8M	ImageNet
ResNet50	25.6M	
ResNet101	44.7M	
EfficientNet-B0	5.3M	
EfficientNet-B1	7.8M	ImageNet
EfficientNet-B2	9.2M	
CNN6	4.8M	
CNN10	5.2M	AudioSet
CNN14	80.7M	
AST (CE)	87.7M	ImageNet + AudioSet
AST (Patch-Mix)		

**Fig. 5:**
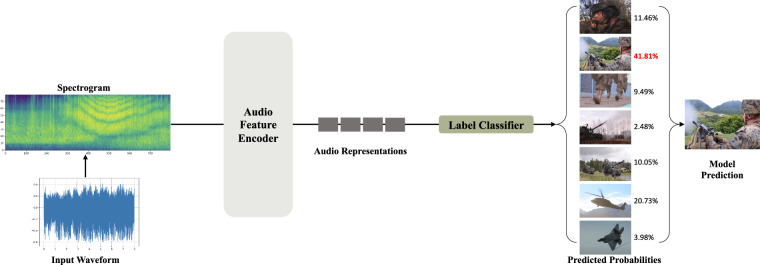
Illustration of overall deep learning-based audio classification process.

The parameters listed in Table 3 represent the number of neurons in the deep learning models, with higher values generally indicating increased complexity and training time. Consequently, the CNN6 model exhibits the lowest parameter count of 4.8M, while the AST model possesses the highest, reaching 87.7M. Fig. 5 depicts the overall audio classification pipeline using deep learning models. In other words, the architectures presented in Table 3 can serve as audio feature encoders, and an additional linear layer acts as a classifier for 7-class audio classification.

### Experimental setting

To prevent overfitting, we employed SpecAugment^36^ augmentation with a maximum mask length of 50 frames and 20 bins applied twice in both the time and frequency domains, respectively. The average value of the given spectrogram was used for masking during SpecAugment implementation. For the AST^10,34^ model, the recommended mean and standard deviation values of –4.27 and 4.57 were employed. The audio classification model was trained using various architectures with cosine scheduling and the Adam optimizer. Except when training the AST model, the model was trained with a learning rate of 1e–3, a batch size of 64, and a maximum of 400 epochs. For AST model training, a learning rate of 5e–5, a batch size of 8, and a maximum of 50 epochs were employed. Momentum update with a coefficient of 0.5 and a decay rate of 1e–6 is applied to all learnable parameters to ensure stable training.

The software environment for conducting our experiments comprised Python version 3.8, CUDA version 11.3, Pytorch, and TorchAudio version 2.0, operating on Ubuntu 18.04 OS. NVIDIA TITAN RTX 24GB GPU is used for AI model training. Considering the potential impact of data training order on AI model performance, five seeds were fixed, and results were reported as averages across these seeds.

### Experimental results

As summarized in Table 4, we conducted an overall comparison of the MAD dataset for the audio sound classification task. We trained various deep learning models with pretrained weights and training from scratch (i.e., without pretrained) using the MAD training set respectively, and evaluated their performance on the MAD test set. For models trained with pretrained weights, accuracy ranged from 88.04% to 91.07%. Among these, the AST-Patch-Mix model achieved the highest accuracy on the MAD dataset. The findings generally indicate a positive correlation between model parameter count and accuracy, the higher the parameters of the AI model, the better the performance. Interestingly, the CNN10 model with 5.2M parameters achieved 89.53% accuracy (0.5% lower than AST), despite having only 6% of the AST model’s parameter count. Conversely, ResNet101, with 44.7M parameters, demonstrated performance inferior to EfficientNet families, as well as CNN6 and CNN10 models with less than 10M parameters.

**Table 4:** A comprehensive comparison of various deep learning-based architectures was conducted to assess their performance on the MAD dataset for audio classification.

architecture	parameters	pretrain	accuracy (%)	
			pretrained weights	scratch
ResNet18	11.7M		88.04_±0.59_	86.83_±0.38_
ResNet34	21.8M	ImageNet	88.27_±0.29_	87.07_±0.74_
ResNet50	25.6M		88.24_±0.41_	86.51_±0.38_
ResNet101	44.7M		88.62_±0.31_	86.86_±0.60_
EfficientNet-B0	5.3M		88.92_±0.17_	87.32_±1.00_
EfficientNet-B1	7.8M	ImageNet	88.85_±0.28_	87.71_±0.33_
EfficientNet-B2	9.2M		89.11_±0.64_	88.02_±0.27_
CNN6	4.8M		89.53_±0.26_	87.57_±0.81_
CNN10	5.2M	AudioSet	90.76_±0.47_	${\underline{89.03}}_{\pm 0.37}$
CNN14	80.7M		${\underline{90.97}}_{\pm 0.34}$	**89.47**_±0.22_
AST	87.7M	ImageNet + AudioSet	90.03_±0.23_	81.17_±0.77_
AST-Patch-Mix		ImageNet + AudioSet	**91.07**_±0.19_	82.20_±1.13_

Employing pretrained model weights from the ImageNet^42^ and AudioSet^29^ datasets is denoted as *pretrained weights*, while not using pretrained weights is denoted as *scratch*, respectively. **Best** and second best results.

In contrast, when models were trained from scratch, their accuracy ranged from 81.17% to 89.47%. The CNN14 model with 80.7M parameters achieved the highest performance in this setting. Surprisingly, the AST model families, despite having the largest number of parameters and being trained on two large-scale datasets, exhibited the lowest performance.

**Fig. 6:**
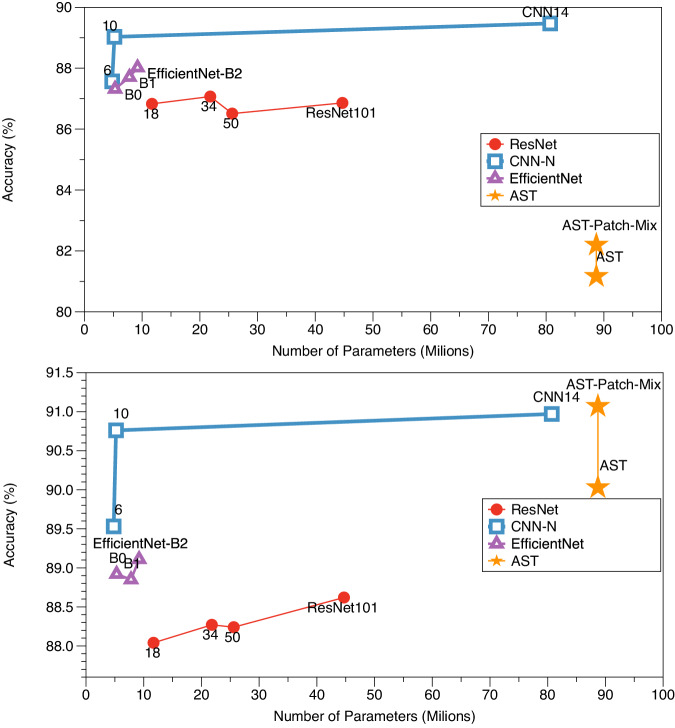
Comparison of pretrained and training from scratch approaches for audio classification – All models were either pretrained on ImageNet^42^, AudioSet^29^, or both, or trained from scratch and fine-tuned on the MAD dataset.

Fig. 6 offers a more intuitive visualization of the aforementioned results. Employing the pretrained weights leads to the highest performance, particularly for the AST model, while training from scratch yields the lowest results. This trend is evident across the range of model parameters evaluated. Consequently, employing pre-trained weights generally leads to higher performance, but at the expense of longer training times. Researchers can select the most appropriate approach based on the specific requirements and constraints of their project.

## Discussion

In this section, we discuss the potential limitations of the proposed MAD dataset.

### Limited scope

While the dataset covers seven categories related to military environments, there may be scenarios or sounds not adequately represented in the dataset, potentially limiting its applicability to real-world situations. For instance, the sound of a nuclear explosion and that of a dive engagement at sea as well as the drone could be included. To the best of our knowledge, this kind of category is hard to obtain, therefore we will regularly update this category of data to the audio community in the future.

#### Limited size

Although our proposed dataset is large-scale data related to the military environment, with 8,075 sound samples totaling approximately 12 hours of audio is not very big. This may be taken into consideration relatively small compared to some other audio datasets, such as AudioSet^29^ and FSD datasets^14^. To address this, we plan to encourage other researchers to overcome the limited number of data issues by deep learning-based modeling.

### Hardware information missing

Although the proposed MAD dataset is military environment data, we are interested in identifying dangerous situations for the general public in our lives. Therefore, we aim to construct the characteristics of audio events with data from various recording devices to identify dangerous situations in a device-independent manner. In addition, we can easily find these trends in the recent papers or datasets that collected from YouTube, which has been widely used in the recent audio or video classification domains. They are also collected regardless of recording device characteristics. Unfortunately, YouTube videos typically lack information about the recording hardware information, resulting in a common limitation for datasets built from publicly available online sources^13,14,29,37–40^.

In that respect, we aimed to improve the audio event classification performance regardless of recording equipment. The reason for this is that our dataset is intended to help ordinary people identify dangerous situations, and even in the real world, audio information comes from different devices, and we cannot handle all of them. Therefore, we designed our dataset to be device-agnostic from an AGI (Artificial General Intelligence) perspective.

### Noise in shelling audio

While the absence of frequency response and the hardware information for the recording devices limits a detailed analysis of shelling sounds, the MAD dataset remains valuable for training classifiers that utilize time-domain features, as demonstrated in Table 4. We believe that further research focused on controlling environments with calibrated microphones could provide a deeper understanding of shelling noise characteristics.

### Audio compression

We acknowledge that the .mp4 format used by YouTube employs lossy compression, which can potentially impact the temporal characteristics of the audio data. However, recent audio and speech, as well as video datasets^13,14,29,37–40^ derived from YouTube are generally deployed with .mp4 or .wav as well as .avi format, and various researchers mainly have used these datasets. In that respect, we believe that its potential effects on the classification model are not critical.

By acknowledging these limitations and outlining potential future improvements, we hope to demonstrate transparency and a strong understanding of the MAD dataset’s characteristics. We believe that this will ultimately strengthen the overall value of the dataset for researchers working on acoustic scene classification in military environments.

## Usage Notes

Our proposed MAD dataset offers a comprehensive collection of real-world acoustic event recordings related to military activities, spanning a wide range of hazard types and scenarios. In other words, the MAD dataset has the potential to serve as a valuable resource for evaluating the performance of existing algorithms and for fostering advancements in the field of acoustic-based hazard detection systems. Besides, the MAD dataset can be utilized for training the AI model which can detecting unidentified objects, explosions, combat objects, and other sound-based hazards, thereby enhancing situational awareness and enabling effective countermeasures against potential threats. By providing researchers with access to this diverse and high-quality data, the MAD dataset facilitates the development and evaluation of more robust and effective acoustic hazard detection algorithms.

## Data Availability

The complete source code and script files employed for format conversion, refinement, and pre-processing of the MAD dataset, as well as deep learning training, are readily available at https://github.com/kaen2891/military_audio_dataset. The majority of libraries and frameworks employed in the code include Python, PyTorch, TorchAudio, Librosa, and Numpy. To execute the code, please follow the instructions provided on the website. Besides, pretrained model weights for each model based on the MAD dataset are publicly accessible. This facilitates researchers in effortlessly loading and employing the AI model corresponding to the performance presented in the paper.
